# From Physically Based to Generative Models: A Survey on Underwater Image Synthesis Techniques

**DOI:** 10.3390/jimaging11050161

**Published:** 2025-05-19

**Authors:** Lucas Amparo Barbosa, Antonio Lopes Apolinario

**Affiliations:** Institute of Computing, Federal University of Bahia, Salvador 40170-110, Brazil; antonio.apolinario@ufba.br

**Keywords:** underwater imagery, computer vision, physically based rendering, generative models

## Abstract

The underwater world has gained significant attention in research in recent years, particularly in the context of ocean exploration. Images serve as a valuable data source for underwater tasks, but they face several issues related to light behavior in this environment. Given the complexity of capturing data from the sea and the large variability of environmental components (depth, distance, suspended particles, turbidity, etc.), synthesized underwater scenes can provide relevant data to improve image processing algorithms and computer vision tasks. The main goal of this survey is to summarize techniques to underwater image synthesis, their contributions and correlations, and to highlight further directions and opportunities in this research domain.

## 1. Introduction

**Figure 1:**
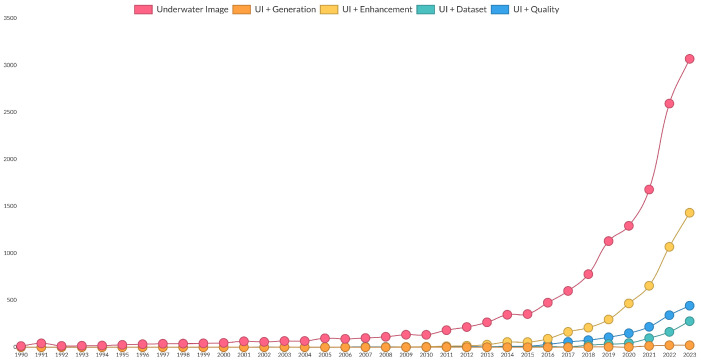
Trends for the keyword underwater image and related tasks. Source: Dimensions AI.

The underwater environment has gained increasing relevance, becoming a research focus for teams worldwide. The chart presented in Figure 1, based on Dimensions AI, illustrates the increased occurrence of the term underwater image in scientific publications.

One of the factors driving this growth is the use of unmanned underwater vehicles (UUV), often operated remotely, and their autonomous equivalents (AUV). These vehicles are equipped with software systems that enable the processing of environmental data for navigation, obstacle avoidance, and task execution [1]. Cameras, when adapted to the conditions, serve as sensors for data acquisition.

**Figure 2:**
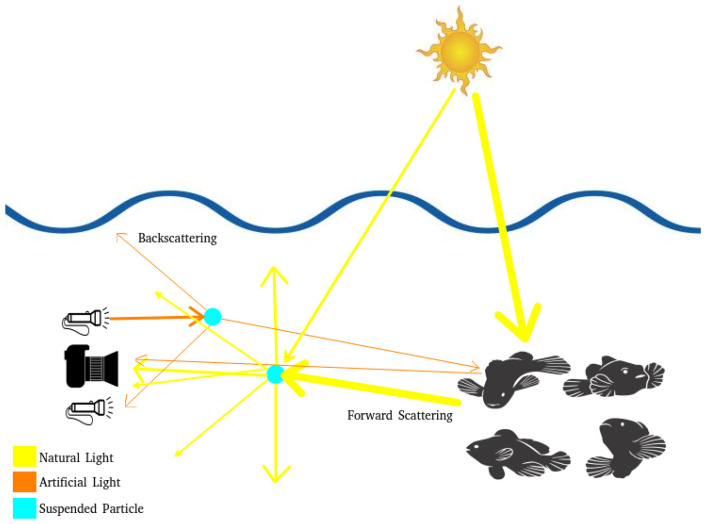
Scattering illustration for underwater environments. Sun, fishes, camera, and flashlight icons from Freepik.

Figure 2 illustrates the image capture pipeline faced by the mentioned vehicles in the underwater environment, which presents several challenges. A significant problem is depth, as even in clear water, natural light is attenuated, resulting in low-light conditions. To mitigate this effect, deep-water operations require artificial illumination. Depending on the environmental composition, suspended particles can deflect light rays (natural or artificial), causing scattering. Artificial lighting can exacerbate the problem, producing backscattering and unwanted reflections in images.

**Figure 3:**
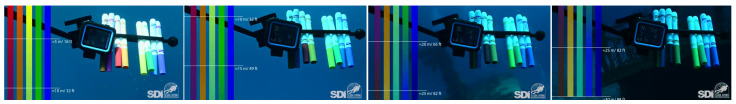
Frames from the video published by SDI on YouTube.

All of these factors can distort the captured images, deviating them from the actual characteristics of the scene. The colors recorded by the sensor are altered as light waves are modified by the environment. Figure 3, retrieved from the video published by the Scuba Diving International (SDI) on their YouTube channel [2] demonstrates this phenomenon.

Researchers have been proposing several techniques to address these challenges, retrieving information from raw data, making the imagery system more stable and reliable to meet the demands. Examples of tasks that would be executed underwater include image enhancement and color correction to improve visibility, as well as object classification, detection, and target tracking.

Even with the evolution of underwater image processing, the high variation caused by the light interaction with environmental elements (also known as participant media [3]) and the complexity of capturing data (diving issues, meteorological conditions, expensive costs, etc.) results in a scarcity of datasets available to address underwater imaging issues.

To address the low data availability, the research community commonly uses simulated data. The recent research has achieved real-world realism on synthetic data, producing larger datasets and enabling the validation and evolution of previous methods to multiple tasks, including image enhancement or object detection.

The main objective of this paper is to provide in-depth analysis of the recent and relevant works about image synthesis for underwater scenes, presenting potential open research points and future challenges for this interesting area. To reach this goal, we present the following:

- A discussion of the current physical underwater image models;
- Selected papers’ relationships and relevance in a graph-based visualization, to enlighten the research trends;
- Further directions for the research area.

## 2. Image Formation Basics

According to Szeliski (2022) [4], “the process of image formation is fundamental to computer vision because it establishes the relationship between the 3D world and the 2D images, which serve as input to computational algorithms. This includes the principles of geometric optics, such as perspective projection, as well as factors like illumination, reflection, and camera sensor properties”.

The rendering equation developed by [5] models the light behavior in discrete surfaces. Based in the incoming $L_{i}$ and $L_{e}$ from the surface, the rendering equation is stated as

(1) $$L_{o}\left(x,\omega_{o}\right)=L_{e}\left(x,\omega_{o}\right)+\int_{\Omega }f_{r}\left(x,\omega_{i},\omega_{o}\right)L_{i}\left(x,\omega i\right)cos\left(\theta \right)d\omega_{i}$$

In this definition, all the *L* coefficients are related to radiance, in a surface (*x*) and a direction ω. $L_{e}$ is the radiance emitted by the surface at point *x* in the direction $\omega_{o}$.

The out-coming radiance $L_{o}$ is composed by the sum of all lights reaching point *x* from domain Ω, calculated using $f_{r}\left(x,\omega_{i},\omega o\right)$, a reflectance function from incoming direction $\omega_{i}$ and out-coming direction $\omega_{o}$. An attenuation is considered by the angle θ between surface orientation, represented by the normal vector in *x* and the incoming direction $\omega_{i}$.

Equation (1) can represent light in a typical environment without considering other light-related phenomena. Any object that obstructs the light will be captured by the camera. However, the nature of the obstruction varies between a solid wall and smoke. This variation is influenced by the size, material, and interaction of particles with light. Small particles, such as those found in smoke, occlude the light passage. Fog and rain produce scattering and increase absorption. The medium containing such particles is referred to as the participant media.

For small particles, such as those in smoke and fog, the environment is referred to as participant media. The effect of these particles must be considered in the rendering equation.

To address this situation, the volume rendering equation extended the rendering equation to deal with participant media an all new effects. Similar to Kajiya, the model calculates the radiance in a location *x* under direction $\omega_{i}$ as

(2) $$\begin{array}{c} L_{o}\left(x,\omega_{o}\right)=-\sigma_{a}\left(x\right)L_{o}\left(x,\omega_{o}\right)+\sigma_{e}\left(x\right)L_{e}\left(x,\omega_{o}\right)\\ +\sigma_{s}\left(x\right)\int_{\Omega }f_{r}\left(x,\omega_{i},\omega_{o}\right)L_{i}\left(x,\omega_{i}\right)cos\left(\theta \right)d\omega_{i} \end{array}$$

That representation considers three relevant effects:

- Absorption ($\sigma_{a}$): when the light is captured by the medium, reducing its intensity;
- Emission ($\sigma_{e}$): when light is generated in the volume, adding energy (and intensity) to the ray;
- Scattering ($\sigma_{s}$): when the light collides with particles in the medium and changes direction, without absorption.

**Figure 4:**
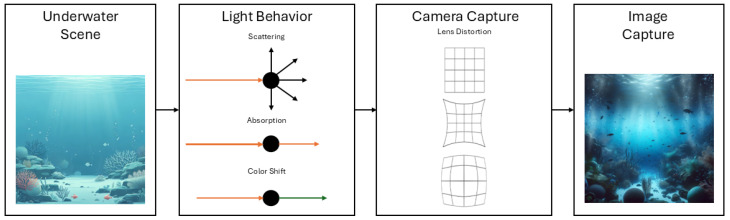
Underwater scene basic capture pipeline. Images generated with Microsoft Copilot.

The main difference between images captured in terrestrial and underwater environments lies in the medium itself. Figure 4 presents the three light behaviors in the basic image capture pipeline for underwater scenes. Compared to in-air medium, the additional behavior is the color shift; based on the absorption of light wavelengths, directly related to the water-line depth, the colors can be seen in a different spectrum. Near-shore waters are influenced by high levels of chlorophyll from plankton. Beach face waters present high levels of particulate matter and dissolved substances. Both cases result in color shifts and turbidity but with differences in visual. Offshore waters can experience high absorption due to depth [6].

The pioneering works of Nils Jerlov [7] evaluated the optical properties of oceanic water worldwide, classifying it into 10 classes based on visibility and transparency. Williamson and Hollins [8] utilized this classification, enhancing the range based on the influence of depth on water visibility and its relevance to underwater processes, such as photosynthesis and deep-water navigation.

Most works on rendering for participating media are focus on homogeneous media and are generally implemented to simulate atmospheric phenomena, such as fog and smoke. When applied to underwater scenes, these techniques exhibit a significant decrease in quality due to the high variability of water types, turbidity levels, and suspended particles types [9].

### Underwater Image Data

In an attempt to compose a computational underwater image model, early works utilized the proposals of McGlamery [10] and Jaffe [11]. These two distinct works are interrelated and are thus considered by the literature to be a single model, known as the Jaffe–McGlamery. In this model, the light received ($E_{t})$ by the camera can be decomposed into three parts: the light reflected directly by the target object $\left(E_{d}\right)$, the scattered light in the forward direction $\left(E_{fs}\right)$, and the backscattered light by particles $\left(E_{bs}\right)$, following the equation

(3) $$E_{t}=E_{d}+E_{fs}+E_{bs}.$$

This model was validated and improved by Blasinski and Farrell [12]. The coefficients are validated by comparing a simulated color chart reference, also known as the Macbeth Chart, with a real one captured in the underwater environment. The model can describe the light interaction with high precision. However, other elements, such as the attenuation component, are not presented in the equation.

Chiang and Chen [13] presented a new model based on the simplified hazy image formation model, commonly used for in-air images:

(4) $$I_{\lambda }\left(x\right)=J_{\lambda }\cdot t_{\lambda }\left(x\right)+\left(1-t_{\lambda }\left(x\right)\right)\cdot B_{\lambda }$$

where *x* is a point in the scene, $I_{\lambda }\left(x\right)$ is the captured image, and $J_{\lambda }\left(x\right)$ is the radiance at point x. The main difference for the model presented in Equation (3) is the function $t_{\lambda }\left(x\right)$, called residual energy ratio, which is related to the wavelength λ and the distance between the camera and the object. For environments without energy loss, $t_{\lambda }\left(x\right)=1$. So, the scattering element $B_{\lambda }$ will be canceled, and $I_{\lambda }\left(x\right)=J_{\lambda }$, representing a capture without degradation.

Akkaynak and Treibitz [14] present a revised underwater image formation model, similar to [13]. The authors add more variables to the image captured by cameras: natural light absorption depending on water depth, scattering based on the type of material suspended in the medium, radiance dispersion based on the distance for the capture target, and more. The condensed form of their model is described by

(5) $$I_{c}=J_{c}\cdot e^{-\beta_{c}^{D}\left(v_{D}\right)*z}+B_{c}^{\infty }\cdot \left(1-e^{-\beta_{c}^{B}\left(v_{B}\right)*z}\right)$$

where the depth of the object related to the water line (*z*) is considered to compose the unattenuated image $J_{c}$, based on the irradiance signal and sensor response and the coefficients for direct light $\beta_{c}^{D}$ and backscattered light $\beta_{c}^{B}$, represented by the vectors v. The direct light **v_D_** is composed of elements such as reflectance and object distance from the camera. The scattered light **v_B_** is composed of absorption coefficients. Both consider irradiance, sensor response, and attenuation.

**Figure 5:**
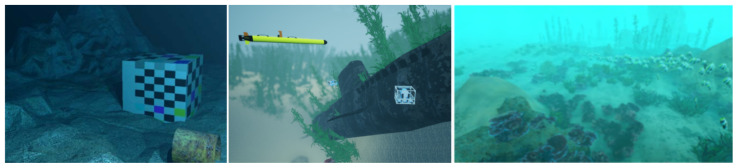
Rendered images from selected works. The first one, from [15], represents the simple physics rendering. The second one, from [16], is an underwater robotic simulator using Unreal Engine to help the rendering calculation. The last one, from [17], is a synthetic AI-based sample created to train a network to remove haze from underwater samples.

Figure 5 represent samples of issues presented in the model, resulting in different kind of degraded image. Researchers have proposed many strategies to address these challenges. However, a strong requirement for the majority of these works is real data, or data that closely resemble real-world conditions. Regardless of the method developed, underwater images are needed for several important purposes: validating physics models, training restoration networks, learning light behavior to generate synthetic data, and more.

Based on the understanding of light behavior in underwater environments, images are identified as a valuable source of information, capturing various elements from these settings. However, according to the elements presented in Equation (5), the captured image can suffer from degradation. The high variability of these environments must be addressed by research teams to achieve significant advancements in this field, even with the challenging situation to capture these data. It is relevant to understand how the teams address the scarcity of diverse data, the techniques they have applied, and the results they have achieved.

## 3. Survey’s Methodology

The purpose of this study was to identify the most relevant scientific works in the field of underwater image synthesis within the major scientific databases. Based on the analysis of these studies, we aimed to determine the most commonly used techniques, the extent of their results, the degree of success in addressing the various challenges in this field, and to identify research opportunities and future directions that could significantly impact the field.

Given these objectives and following the classification proposed by Grant and Booth [18], this work is a scoping review that uses the following steps as its methodology: database search, scope definition, papers selection, refinement based on abstracts, and organization of the final selection of paper to be analyzed in the review. The following sections will present each of these steps in deeper detail.

### 3.1. Research Databases Selection

Multiple online databases are available to find relevant publications in computer science research. This study used the following databases, considering its relevance and coverage: IEEExplore, Scopus, MDPI, and Google Scholar. Additionally, ArXiV is used to find early access papers and publications from non-open access platforms. The selected papers are limited to the year of 2023.

### 3.2. Scope Definition

The keyword “Underwater Image” was chosen as the primary search term. An initial filtering process was applied to refine the results. For instance, studies mentioning “underwater image” in the context of marine snow chemistry were excluded, as they do not align with the study’s focus. This review specifically targets research on underwater images within the domain of computer vision.

**Figure 6:**
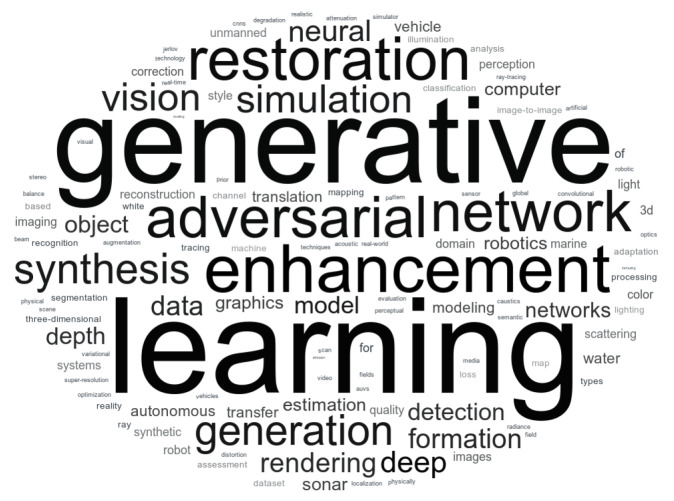
Word Cloud from keywords retrieved in selected papers.

After the initial filtering, we selected 101 papers. To analyze the key themes within the selected papers, we extracted the keywords provided by the authors and used them to generate a word cloud. This visualization highlights the most frequently occurring terms, offering insights into the dominant topics within the reviewed literature. By emphasizing recurring keywords, the word cloud helps illustrate the central research areas and trends in underwater image analysis within the computer vision domain. The word cloud shown in Figure 6 highlights the most frequent terms from the collection.

From this analysis, the words generative (variant generation) and enhancement (variant restoration) appear in a significant number of works. Rendering techniques are also notable, with terms such as learning, simulation, and synthesis frequently appearing. The correlation between the published papers can be highlighted by the keywords and citations. This analysis can be used to identify trends, gaps, and contributions. It is possible to present the most relevant works for the research purpose and techniques groups from the selected set.

### 3.3. Research Analysis

To analyze the correlation between publications a graph representation was produced. Each node represents an article, and each edge represents a citation between the works.

The number of citations was used to weight each node of the graph, which changes the node size. Two citation counting metrics were used to define these weights: “General Citation” represents the count from any publication, retrieved from Google Scholar. The “Inner Citation” is defined by the count among the selected papers.

The representation of the class of nodes is based on a category, selected using the work’s keywords as references, capable of describing the main objective of the article. For example, multiple publications use “generation”, “generative”, and “synthesis”. All these papers can be grouped in the category “generation”. The categories can be described as follows:

- Generation: works that produce some data;
- Enhancement: works that improve some feature from captured data;
- Rendering: works that compute an image using geometric elements;
- Dataset: works that produce a categorized collection of data;
- Detection: works that execute the task of object detection;
- Quality: works that calculate quality measures for data;
- Review: works that execute a topic review in the research area;
- Model: works that propose the image generation models;
- Superresolution: works that increment image resolution for data.

**Figure 7:**
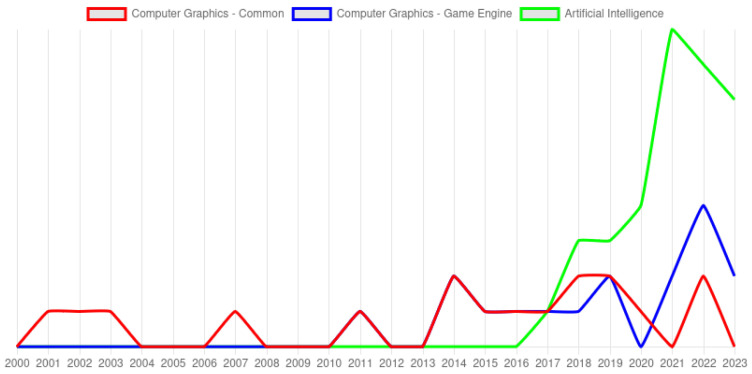
Publications separated by years. The growth of AI papers after 2016 could be explained by the success of CNNs, GANs, and similar architectures.

It is worth noting the high presence of generation and enhancement works. Multiple papers found in the search used a generative adversarial network (GAN) [19] to enhance the captured data. Based on Figure 6, “generative”, “adversarial”, and “network” are prominently featured. The most recent works, as presented in Figure 7, use artificial intelligence (AI) to learn underwater image features and address related issues.

Using this insight as a guide, it is possible to understand the relevance of data collections in this context. In the collection, the most-cited paper is a dataset publication connected with all the other keyword clusters. Assuming the real-world data capture process is not the objective of this survey, a second graph was produced detailing the computational techniques used in each selected work. If a paper employs AI to achieve its objectives, it is labeled as ‘AI’. If a paper uses physically based synthesis models, it is labeled as ‘CG’. If no relevant techniques are used, it is labeled as ‘None’.

### 3.4. Abstract-Based Filter

In the next step, each abstract was reviewed to assess adherence to the proposed review. Many works address image enhancement, even using synthetic data to train the model or validate the algorithm. However, few produce synthetic data. Techniques to solve other issues, such as color correction or haze removal, are also presented. These works are captured by the keywords because the “underwater image” filter is used in the context.

**Figure 8:**
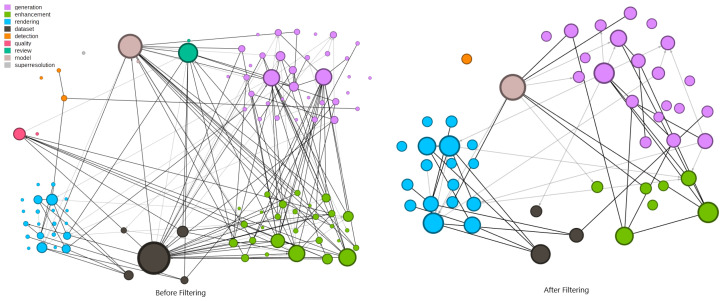
Graph generated from selected papers. On the left, the full collection. On the right, after the cleaning task. The reduction in nodes is related to the presence of data generation in the published work. Node size scaled by min–max method.

Additionally, if the data representation used in the paper is not similar to those produced by real-world cameras, the publication is removed from the set. Exemplary works include sonar-produced images or volumetric and depth data. Review papers are also excluded as they do not provide original data for analysis. This filtering process resulted in the removal of 51 papers from the collection. A comparison between the first graph version and their correspondent after filter is presented in Figure 8.

### 3.5. After Filtering Discussion

Figure 8 shows the new configuration of nodes and edges of the citation graph after the complete filtering process. It was executed in two steps.

The first was the removal of dataset-only papers (highly cited) on the graph, based on the absence of data generation in the publication. Real-world captures or synthetic collections without an explanation about how the images are generated were not useful in this context. Furthermore, enhancement papers are excluded if they did not involve the generation of synthetic data, as they were not pertinent to our purpose. Many papers on this topic cite other research to compare results.

It is important to note the presence of outliers in both versions. The most relevant papers maintain their significance after the removal. Papers with low citation counts are removed without significantly impacting the relationships. A few publications are still presented even without direct connection to other selected papers, as they introduce methods used in the generation of synthetic data.

**Figure 9:**
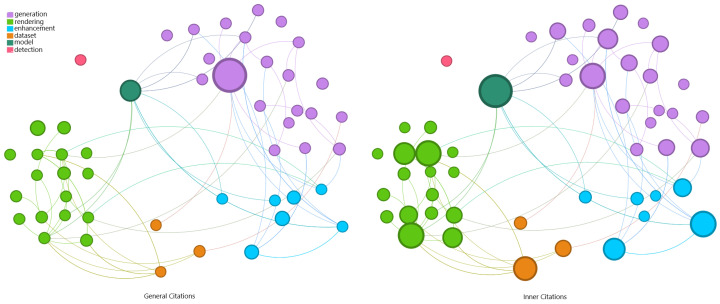
Graph representation for single word definition on the remaining papers. The synthesis task is notable, captured by the categories of rendering and generation. Node size scaled by min–max method.

Figure 9 illustrates the significant presence of publications related to generation and rendering. All other categories combined do not exhibit the same level of representativeness as either of these two categories individually. This trend highlights the need for new underwater data to validate computer vision tasks and address the lack of diverse information.

**Figure 10:**
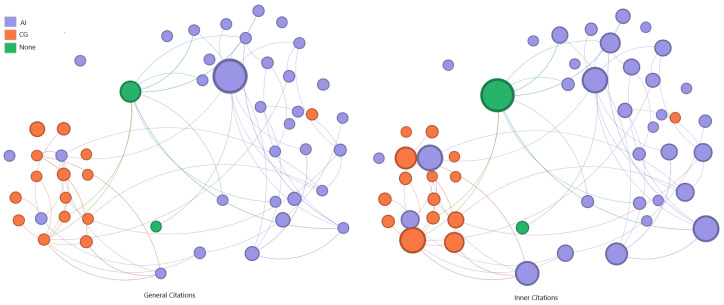
Graph representation for the technique used on the remaining papers. The correlation between the category from the previous graph and the technique applied on the publication is significant. Node size scaled by min–max method.

If the generated data are relevant in the selected works, the technique applied in this process is equally important. In Figure 10, the nodes (papers) are positioned similarly to the previous graph. The correlation between generation and AI is remarkable, as is the correlation between rendering and computer graphics.

Based on the data and correlations presented, particularly in the graph in Figure 10, the articles were grouped by the image synthesis technique:

- Physics-based Underwater Image Generation:This group holds papers that used physically based models to compute the light path and its interactions with the participant medium and computer graphic techniques to produce realistic underwater images;
- AI-based Underwater Image Generation: In this group, AI models were used to learn how underwater images look and to produce new images replicating the trained data.

**Figure 11:**
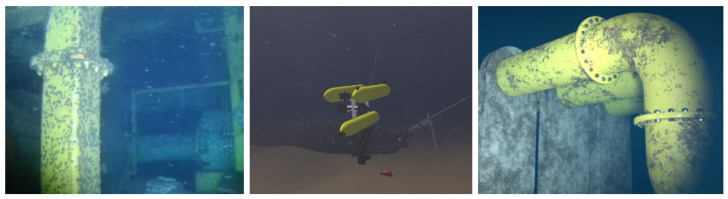
Comparison between real-world (first image), simulation with cartoon visual (middle image) and realistic visual (last image). First and last image from [20]. Middle image from [21].

Considering that the results of the selected papers are images, there is a definition of realism. In this study, visual realism or realistic image is defined as the perceptual similarity of generated data to real-world photographs, as evaluated by human observers. In contrast, non-realistic images lack this similarity. An example is a visual similar to a cartoon, with simplified textures and reduced detailing. Figure 11 shows a visual comparison based on this definition.

After the definitions and graph analysis, the following sections present a resume of the selected works.

## 4. Physics-Based Underwater Image Generation

**Figure 12:**
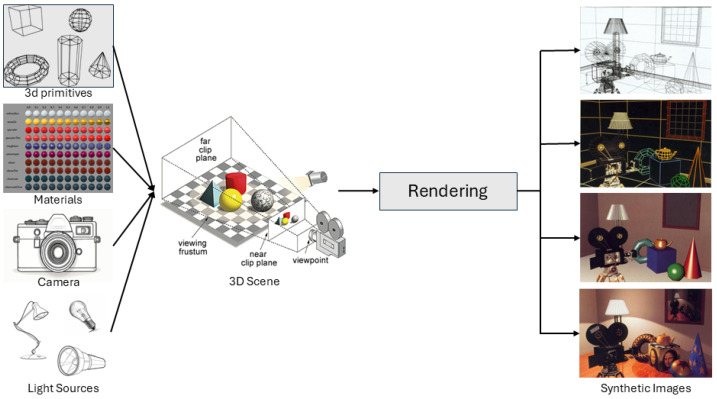
Computer Graphic Rendering Pipeline. Scene and Camera parameters are useful to improve realism in the rendered image. Rendering images from [22].

For papers that use physics-based techniques (Figure 12), two paths can be defined: the first path involves publications that used only physics models to emulate the behavior of light in underwater environments. In this case, the developer needs to implement all the light model using a programming language; The second path involves using a rendering framework, usually a game engine. This tool can minimize the effort in the model implementation trough standardized functions and validated libraries.

To produce high-fidelity samples, the rendering strategy requires a physical accuracy, utilizing an improved physical model. Consequently, the rendered image can exhibit shadows, light absorption, scattering, and other effects. However, this advancement increases algorithmic complexity and resource consumption. The use of a graphic processor unit (GPU) has enabled the development of more accurate models, reducing process execution time.

Jensen and Goliáš (2001) [23] proposed a rendering pipeline for behavior in deep water, combining state-of-the-art methods at the time, including waves, turbulence, and surface tension. The waves are simulated using the Fourier transform as data source statistics from oceanography. To render foam and water spray, Newtonian models are implemented with few changes in the pixel transparency to adapt the size of the rendered object. The water physics interaction is computed respecting real-world physics. Even with the focus on surface rendering, the publication brings improvements for underwater visualization.

Iwasaki, Dobashi, and Nishita (2002) [24] propose using a GPU to accelerate the computation of optical effects in an underwater environment, reaching up to 22× faster processing than using typical GPUs. Even with this increment in time, a limitation of this work is related to the simple scenes used in the examples.

Bo, Kai, and Cui (2007) [25] improved the method presented by Iwasaki [24] using an eye-irrelative rendering and volume-based shadow computation. The results achieved a better frame rate even using no GPU acceleration. However, the visualization is not realistic, looking similar to a cartoon.

Cerezo and Seron (2003) [26] presented a system capable of generating information about illumination and colors in underwater ambiance, solving the issue related to light transport in an anisotropic (the different light behavior for different surfaces) environment and with multiple scattering. The work uses the discrete ordinate method for light calculation, computing the participant media as discrete volumes, resulting in a fast algorithm for simple scenes. However, the results in the paper show that rendering is highly dependent on a good spectral coefficient selection, demanding a careful choice of absorption, emission, and scattering coefficients to represent the environment.

A relevant model used by the authors is presented by Jaffe [11] and McGlamery [10]. Usually known as Jaffe–McGlamery, this model can address the underwater light effects, such as absorption and scattering.

Sedlazeck and Koch (2011) [15] proposed a simulator for light behavior using Jaffe–McGlamery’s underwater imagery model. Another relevant feature of this publication is the light refraction in the camera underwater housing, custom structures to turn common cameras useful for underwater scenarios. These protections usually had glass in front of the camera lens, producing changes in the index of refraction [27] and the image captured.

Allais et al. (2011) [28] developed a simulator, called SOFI, to generate synthetic underwater images. The execution is in near real time, using GPU programming with CUDA. The tool can be used through a graphic interface, which allows the scene configuration without programming effort. The degradation for simulated images in underwater environments is computed using the model developed by McGlamery and Jaffe [10,11].

Stephan and Beyerer (2014) [29] addressed the challenges associated with underwater imagery, characterized by low quality caused by light interactions with water and particles. The authors propose a physics model for rendering images and a restoration approach based on Jaffe–McGlamery’s model. Degradations like contrast loss, brightness from scattering, blur, and color shift are taken into account in their computation. Three components are considered: direct lights, blur, and indirect lights. The first one computes the light from sources captured by the camera. The blurring is calculated from the light scattered from water and suspended particles. The last component is computed from the reflected light in particles. This backscattering strategy would not be realistic because the multiple reflections were not computed, generating only homogeneous media images with low turbidity impact.

Blasinski and Farrell (2015) [30] addressed the problem related to coral reef decline, caused by CO_2_ increase in the atmosphere and water temperature changes. A relevant source of information for this issue is image captures to classify the pigments from analyzed reefs. To improve the variability for the known captured data, the authors propose a simulated environment, changing multiple variables in the capture: depth, light spectrum, infrared, noise, and more. These data are useful to understand the change in the reef “colors” and inspect the element.

Blasinski et al. (2017) [31] presented a work that deals with the first step of underwater capture: simulate the light behavior on the camera sensor. The physics of light are computed using ray tracing techniques. Real-world images are used to fine tune the simulator parameters, resulting in a real-world similar simulated image. The authors used a Macbeth chart to validate the implementation.

Dudhane et al (2020) [32] propose a technique to produce a more generalist restoration model for underwater images. The authors produced an approach to generate synthetic images to use as a reference in the model’s training step. The image formation equation is adapted to simulate the three most relevant distortions in underwater images: color shift, blur, and light absorption. The output dataset was used to train an AI model to restore images.

Song et al. (2021) [33] presented a work that deals with the lack of data for deep-sea and their reference to be useful in computer vision algorithm tasks. The authors provide a simulation based on the Jaffe–McGlamery [10,11] model with multiple light spots and an analysis of the model’s coefficients, aiming to optimize the performance. As input to the framework, in-air images and the respective depth maps are used to generate synthetic data with underwater optics effects. Jaffe–McGlamery’s [10,11] model deals with light attenuation (absorption and scattering) delivered from many light spots using a single ray direction.

Liu et al. (2022) [34], addressing the issue related to the color shift in underwater images, implemented a degradation model based on Jaffe–McGlamery’s [10,11] underwater imagery equation. The method generates synthetic images with color shift issues similar to real-world data, providing a useful dataset to train their image enhancement AI network.

**Figure 13:**
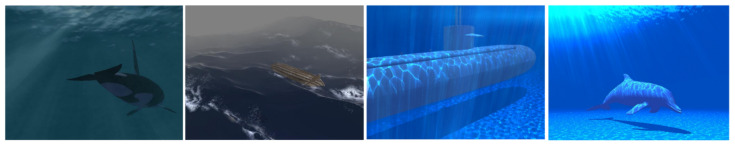
Examples from [23], presenting underwater (first image) and surface (second image) rendering. The last two are from [24], rendering clean underwater scenes.

Recently, many research teams start to use game engines and other computer graphics frameworks to render realistic scenarios, such as Unity or Unreal, OpenSceneGraph, Gazebo, and more. A relevant feature of this rendering is the use of GPU through the rendering libraries (e.g., OpenGL or Vulkan). Figure 13 presents samples of this generation technique.

The engines can provide a high definition image, multiple configurations to capture data, and easy configuration for camera position. In Unity and Unreal, for example, building a complete scene without implementing codes is possible, tanking effort only in the rendering code.

The Gazebo simulator, a relevant tool for robotics development, has a powerful physics computation for movement and body interactions. However, the light behavior is not realistic enough for the current rendering results. Using this framework, Carvalho et al. (2015) [35] developed a virtual reality tool to simulate offshore scenes in the oil and gas industry, including the interaction between robots and structures in the underwater environment. Based on the main goal of the simulation framework, the produced visual data are not realistic, similar to a cartoon.

Doernbach et al. (2018) [36] developed improvements in safety, efficiency, and reliability from robotics operations in the deep sea on the Gazebo simulator. The framework proposed can evaluate the performance and minimize the discrepancies between real-world and simulated data. A simulated stereo camera is implemented, providing depth information in the generated image. This depth is used to compute the light attenuation. The main contribution of this paper is the relationship between the real and simulated image. The real data are helpful to correct the simulated, bringing realism; simulated data can be used to improve the visibility of real data, computing enhancement coefficients.

Alvarez-Tunon et al. (2019) [37], facing issues from marine robotics, presented a framework to develop and improve the quality of the computer vision algorithm pipelines. The lack of data and high condition variability make the execution of the enhancement process difficult. Keeping the use of the Gazebo simulator, for computing light behavior and underwater physics, the framework uses a domain transfer technique to transform the simulated image in the required real-world environment.

Zhang et al. (2022) [38] presented a general robotics simulator using Gazebo and the plugin UUV simulator for underwater physics. The main objective of this work is to provide data simulation (LiDAR, Sonar, etc.), but it can provide visual information too, improved by data distortion provided by the Blender, a tool for 3D drawing and modeling, programming interface.

To improve the rendering quality, simulators can be developed using osgOcean, a tool developed over the OpenSceneGraph that presents an improved marine visual rendering. The work presented by Kermorgant (2014) [21] has as its main objective the physics interactions between underwater vehicles. However, to deliver improved visual elements, the author used osgOcean, with the presence of color, visibility, and floating particles. However, the framework has a simplified version of each feature. The visual results present improvements when compared with older versions, using the Gazebo simulator, but do not achieve a high fidelity realism.

Blender is useful to build the scene elements (rocks, fishes, boats, etc.) and compose elements in a single 3D model. However, the tool can be used to render the images. The VAROS synthetic dataset was presented by Zwilgmeyer et al. (2021) [20] using image captures from 3D scenes built on Blender. The configuration for sampling pixel calculation allows a high-quality rendering but with relevant hardware consumption. The produced data achieve a realistic result.

With the same tool, Lin et al. (2022) [39] developed an Oyster Reef simulator, providing an environment capable of producing realistic data for multiple sensors and enhancing the monitoring of this harvest. This simulator is used by the same team in another publication: Lin et al. (2023) [40] use the data collected in the simulation to train a network model for oyster detection. Palnitkar et al. (2023) [41] combined a large language model (LLM) with the data generation from OysterSIM [39]. The authors understand the scene requirements using the LLM, render the scene using the simulator, and capture data useful for detection tasks.

To achieve more accurate realism in visual rendering, game engines are a useful tool, bringing more power to image generation and useful APIs to make easier programming. Released in 1995, the Unreal Engine is a consolidated tool for game development, particularly when realism and physics-based modeling are required.

Manderson et al. [42] presented an Unreal-Engine-based simulator for their real-world robot: Aqua. In the simulated environment, virtual sensors are implemented to capture data and produce datasets for marine robotics-related tasks, including cameras.

Koreitem et al. [43] used Unreal Engine to produce data useful in the improvement of results for a 3D pose estimation task. High realism is not required for this issue; simplified visual features are sufficient to compute the estimation. Therefore, exponential fog is used to simulate the visual characteristics of underwater environments. Recalling the exponential behavior of the underwater environment image, as in Equation (5), the default exponential fog in game engines serves as a useful approximation but does not achieve realistic imagery.

Thompson, Chalmers, and Rhee (2019) [44] bring the underwater scenes to virtual/mixed reality applications, producing 360º videos rendered in real-time with underwater features (godrays, caustics, fog, and more.). The produced algorithm is compatible with Unity3D and Unreal Engine, easy to integrate into other applications.

Mousavi et al. (2021) [45] presented OpenWaters, an underwater simulation kit produced using Unreal Engine and their in-built ray tracing techniques to improve the realism of the generated data. The synthetic data are used to train a depth estimator deep model, validating their use for computer vision tasks. Extending this work in [17], the authors presented a underwater image enhancement AI-based model trained using images generated by OpenWater.

HoloOcean is a simulator capable of helping the development of autonomous underwater vehicles (AUV), delivering realistic sensors, including an RGB camera [16]. Using Unreal Engine, HoloOcean provided realistic underwater imagery. However, no participant media are implemented, limiting the data to good visibility conditions, such as offshore waters or near water line regions.

Yao et al. (2023) [46] created a new Unreal Engine environment, based on AirSim. The main goal of the simulation is to create data for distance estimation in underwater scenarios that are useful for other computer vision tasks.

UNav-Sim [47] uses the Unreal Engine to create realistic scenarios. Fully integrated with a relevant robotic framework, the simulator has a lot of sensors implemented, providing a lot of data for increasing the development of robotic systems. The sensors include cameras, useful for the visual navigation task validated by the authors. Álvarez-Tuñón, Kanner, and Marner (2023) [48] presented a synthetic dataset collected from four scenes built in that underwater simulator.

Another relevant game engine tool is Unity3D, released in 2005. Compared to Unreal Engine, there are differences in the programming language and user interface, but the overall results for rendering are similar.

Chen et al. (2022) [49] produced a physical interpretable scene simulation using Unity3D. The imaging model implemented by the authors is composed of two components: the model from Jaffe–McGlamery and the foggy degradation model for in-air captures. The water types and the water depth are the main variables from this implementation, computing the color decomposition to estimate a more accurate light attenuation in three spectra from color images.

Loncar et al. (2022) [50] provided the Marine Robotics Unity simulator (MARUS), developed using Unity3D. The main objective of this simulator is to provide data for marine environments, using boats and surface vehicles. However, underwater scenes are implemented, simulating divers or AUV navigation.

In this section, publications using physics-based underwater image synthesis techniques are summarized. By employing direct computer graphics programming or rendering frameworks, the realism and quality of the results have increased as processing power has grown. Refined imagery models and better coefficient definitions have also contributed to this evolution. However, despite achieving high similarity images, the rendering process continues with an elevated computational cost.

To present a simplified and comparative representation of the selected works, Table 1 was compiled. The columns summarize key features extracted from the reviewed publications, allowing for a structured comparison of the rendering techniques employed by different authors. In the context of game engines, the default rendering pipeline is commonly utilized due to its ease of configuration for image generation. However, for enhanced realism, the rendering can be further refined by adjusting parameters or enabling more sophisticated light computation techniques.

Visual features, such as godrays, haze, and color shift, are also remarked, given the relevance to represent a realistic underwater environment. The presence of participant media can increase the data variability produced by rendering. The combination between the visual elements can produce a realistic simulation.

Based on elements presented in Table 1, the use of rendering frameworks is remarkable. Another interesting appointment is the realism achieved by Unreal Engine and Unity3D, as these frameworks are widely used to produce high-fidelity video games. Features related to realistic rendering are readily available. Regarding visual elements, color shift is the most prevalent. Regardless of depth or target distance, the color shift will be present in underwater environments, varying only the intensity.

## 5. AI-Based Underwater Image Generation

Despite the availability of real underwater collections, accurately representing the full variability of underwater environments remains a challenge. The complex interplay between color alterations and diverse types of suspended particles leads to significant variations in light behavior. This variability can be addressed through AI models capable of generating new samples of the target environment by manipulating data derived from initial parameters, textual descriptions, or reference images. Such an approach presents a viable solution for mitigating related data insufficiency.

As mentioned in Section II, many publications use the underwater image model to estimate the light behavior in the environment. For AI models, the strategy can be replicated: with the information about the depth in the captured image, it is possible to estimate the light degradation in the environment and reproduce it underwater, creating new samples and increasing the dataset.

Ueda et al. (2019) [51] provide a network model capable of producing a synthetic underwater image from an in-air common image and their related depth map. The model estimates the light interaction with the environment and the output image can be used to develop restoration techniques.

Wu et al. (2021) [9], proposed a physic-based machine learning model that receives the original image and their respective depth map as input and delivers a distorted simulated underwater image, producing new samples of data. The model is trained using a similarity metric as regularization to keep the relevant information in the output.

Jain et al. (2022) [52], when challenged by color shift issues in underwater images, proposed a method to generate a diverse dataset to represent multiple real-world scenarios by domain adaptation techniques. The rendering pipeline from the method converts in-air images (using the pair color and depth) into underwater. After this, real-world samples are used as data sources to compute coefficients and replicate them in the synthetic data.

Polymenis et al. (2022) [53], addressing the object detection issue related to remotely operated vehicles and their mission targets (pipes and submarine structures), proposed a strategy to convert in-air structure images in underwater samples. The output can increase the size of particular datasets and help to improve models for object detection.

Wen et al. (2023) [54], using a physically guided synthesis model, presents SyreaNet. This framework is planned to execute image enhancement, using a network model trained with real and synthetic data. Synthetic data are generated using a common image and their depth map as input. An image formation equation [14] is used to compute the underwater effect, producing an image pair: clean and degraded.

Wang et al. (2021) [55] created an image enhancement method based on a strategy called analysis-synthesis, which consists of using two architectures. The first one executes an analysis of the underwater image, trying to retrieve prior information about the scene: water type, coefficient estimation, etc. The second one, guided by the priors, executes the enhancement. The authors propose a method to create underwater images from in-air common captures. The synthetic data are produced using three “configurations”: water type, based on Jerlov; depth from captured data; and color from the expected scene. The new dataset includes clean images, their corresponding degraded versions, and the associated coefficients.

If the depth information is not available, an alternative is to use known coefficients from the image formation for a particular underwater environment. In this way, the dataset representativeness can be increased.

Hong, Fulton, and Sattar (2019) [56] faced an issue with scarcity of marine litter captures. The data were required to train an improved detector to be deployed on robotics systems. Thus, to create new examples and increase the data variability in their training set, the authors proposed a variational autoencoder to produce images.

Li et al. (2020) [57] try to deal with the challenging task of capturing underwater images. They proposed a synthesis algorithm based on prior knowledge about the target scene and a physics-based model to calculate light behavior, producing different scenarios and capturing data to train enhancement models.

Desai et al. (2021) [58,59], In an effort to address challenges associated with underwater image restoration, the realistic underwater image generation (RUIG) method was proposed to generate synthetic images suitable for training a conditional GAN. The generated dataset is useful to train a multi-objective enhancement AI model capable of restoring images with different attenuation coefficients, due to the variation added using an equation [14] and the coefficients from Jerlov water types.

Levy et al. (2023) [60] used neural radiance fields to produce an improved network model for scattering media. The rendering model presented by authors is capable of producing new underwater images and their backscattering map. The coefficients learned in the training phase were useful to retrieve clean information for degraded images.

**Figure 14:**
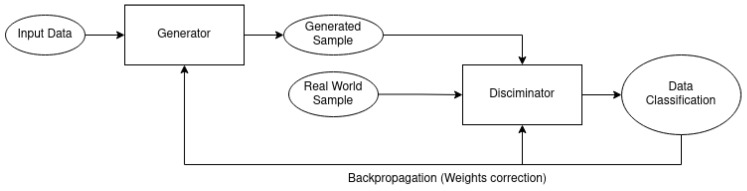
Generative adversarial network (GAN) model utilized to generate samples from noise input data [19].

Even though it is a good strategy, this method has limitations on the generated images’ realism. To address this issue, researchers proposed synthetic data generation. A relevant technique is generative adversarial network (GAN) [19], illustrated in Figure 14, which is capable of generating samples similar to real-world images from a not structured input.

Li et al (2017) [61] proposed the WaterGAN, a GAN model that receives a common image and its depth map and produces a synthetic underwater image. The network was built using multiple generators to estimate component capture: light scattering and attenuation, also camera parameters.

Cheng et al. (2018) [62] proposed a method to improve underwater images, fixing problems such as color shift, contrast balance, and blurring. The last phase of the work applies a GAN model to amplify the image resolution, improving the quality of the input.

Yu et al. (2018) [63] introduced a GAN model capable of restoring underwater images degraded by color shift and bad lighting. The main contribution of this publication is the use of perception loss with adversarial loss. The addition of perceptual loss produces a generation model with better visual quality, once the network weights are trained using high-level features extracted from the outputs and compared to the input.

Zhao et al. (2021) [64], with common image pairs (color and depth), proposed a strategy to synthesize new underwater images, based on color, to create new pairs: a new submarine version and their related depth map. The new images were degraded using the depth data to simulate the light behavior. They extended their work [65] and proposed a dual GAN approach. The first one creates underwater images using the real depth map and in-air captures. The second GAN is responsible for estimating the depth from the synthetic underwater data. The ground-truth is used to refine the training.

Liu et al. (2021) [66] addressed the challenges related to capturing high-quality underwater images. The authors proposed an adversarial network that integrates a physics-based image formation model with the adversarial generation. Using the equation from Akkaynak–Treibitz [14], the training is controlled by these values to converge in a realistic synthetic image. Missing regions could be generated by the model, completing the missing data.

Fathy et al. (2023) [67] try to convert in-air images from common datasets into underwater versions with coherent distortions. The SubmergeStyleGAN applies style transform based on real-world captures in the training stage, producing a very similar output from the generator. However, the results are improved in the inference stage using the depth data from the input image, refining the attenuation estimated values from the generative step. The synthetic data are used to improve image enhancement algorithms.

Galetto and Deng (2023) [68] developed an image translation task to remove marine snow on underwater captures. To achieve this goal, the authors trained a generative model to create a paired dataset degraded by marine snow, inserting synthetic snow into real-world data. The created dataset was used to train the final task model.

**Figure 15:**
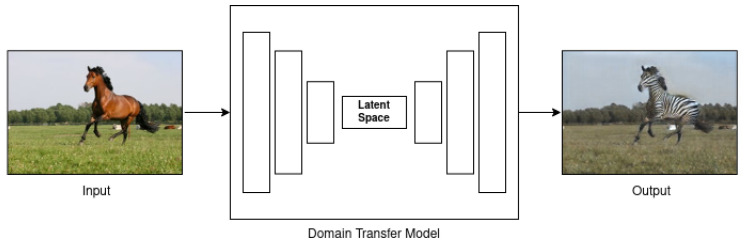
Domain transfer (style transfer) model illustration, used to change the information from the input image in the desired output. Images from [69].

Another way to deal with AI-based synthetic data is called style transfer, when the model learns features from a small dataset and modifies others to transform (see Figure 15) data similar to the trained [69]. It is also called domain transfer or image translation.

Li et al. (2018) [70] deal with the issue related to the absence of ground truth (paired images) for underwater scenes. A network was proposed to transform in-air images in underwater scenes since the model knows the parameters for the required scenario.

Chavez-Galaviz and Mahmoudian (2022) [71] faced issues using convolutional neural network (CNN) in hardware-limited devices, such as embedded devices. Given the absence of data to train the model, the authors also proposed a technique to create synthetic data using style transfer to create new underwater images similar to the real-world dataset. The model is trained using these data.

The techniques presented thus far are the most common strategies for image generation in the underwater domain. However, some works presented other methods to solve the generation of underwater images.

Ahmed et al. (2022) [72] produced a publication capable of transforming in-air images into underwater versions based on Jerlov water types. The method leverages depth information to enhance results; however, it is not a strict requirement. If depth data are unavailable, the image is assumed to be at a maximum depth of 10 meters, and this estimated depth is used to compute the transmission map. All the conversions use statistics computed in a reference dataset, using MATLAB R2024a. The produced result can be understood as a machine learning method.

Ye et al. (2022) [73] considered the complexity and high variability of underwater scenes. Thus, to create a single network architecture capable of providing various underwater types of images, the authors proposed a novel generation strategy: an underwater environment sample is given for the model. From the reference, distortion coefficients (called natural light field by the authors) are extracted. In another way, the generator receives a clean in-air image as input. The depth data from the input are estimated. The input image is transformed into a new underwater sample using a combination of the coefficients and the depth data.

Pergeorelis et al. (2022) [74] faced the semantic segmentation challenges for underwater images. The real-world datasets are limited. The authors proposed a strategy to improve the size and variability of each capture, combining multiple images in a single one. For any images with information (a fish or a diver, i.e.), the segmentation annotation can be used to extract only the target from the original image and inserted in a second one. After the insertion, the produced image is blurred with a Gaussian blur, to smooth the edges from the generated item.

The presented publications were developed using artificial intelligence to deal with the low amount of underwater data. To create new samples or reproduce the behavior of an example in other images, the results for each method were used to improve visual tasks. Many works just used the new samples to reach their goal, i.e., image enhancement. Without access to the synthetic dataset, just the pipeline to make these images is useful for new research.

Similar to Section 4, Table 2 provides a simplified and comparative overview of the selected works. The model column presents the model name, if designed by the authors, or the base architecture used on publication. The columns GenAI and Style Transfer are helpful to understand the AI task developed by the model. The column ’Require Depth’, in this context, means the algorithm executed needs information about distance between the camera and target. Another important feature presented is the validation of the method on real data and in the wild data. The first validation is related to the use of any underwater image data as source data, validation, or comparison with the produced results. In the wild is more representative underwater data collection, captured by authors in multiple underwater sites or using a well-validated dataset, such as UIEB [75] or UID2021 [76].

The features selected for Table 2 are derived from the objectives of selected papers. Most of them are task-related, focused on specific issues such as image enhancement or color correction. Consequently, visual effects are generally absent in the generated data, except for color shift. Many authors used the classification from Jerlov, based on color classes, to organize their synthetic datasets. Even participant media, a significant feature of underwater environments, are not extensively addressed in AI-based techniques. Another notable aspect is validation. Almost all authors validate their methods using real data, captured in controlled environments. However, only a few validated their methods in in-the-wild collections with diverse underwater configurations.

## 6. Discussion and Challenges

Using the selected works as a baseline for understanding the state of the art in underwater image synthesis, a significant observation emerges: The most relevant papers were built using more than one generation strategy.

In the early years of computer graphics, the geometric (and physical) rendering brought a sense of realism to the generated images. The rendering processes executed were expensive and constrained by performance limitations. The inherent parallel processing capabilities presented by GPUs allowed the execution of more complex computations than was previously possible, improving the performance. After the popularization of parallel computing frameworks (CUDA, e.g.), physics-based rendering became the most prominent method for generating realistic underwater images.

**Figure 16:**
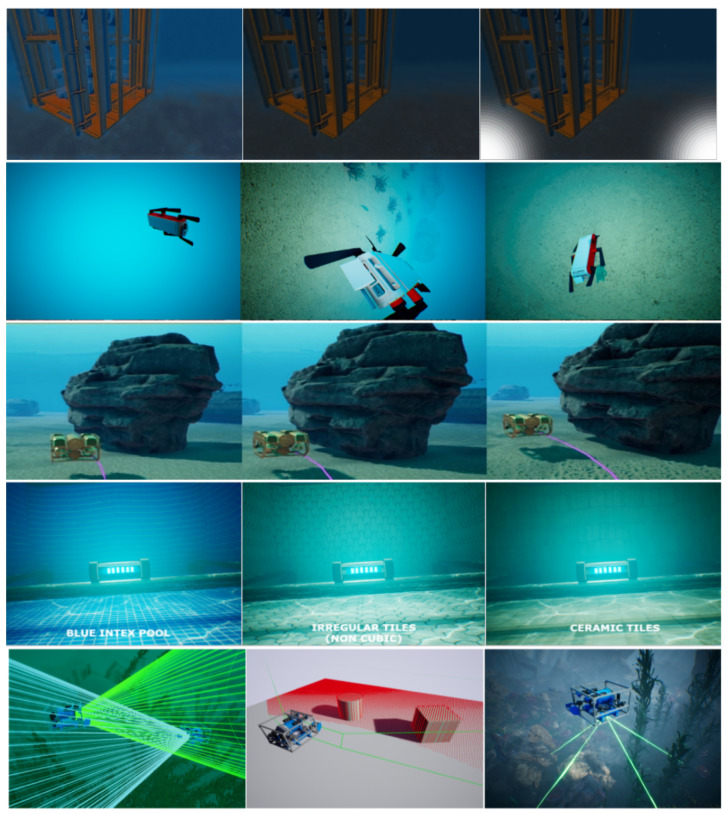
The first row presents the results from Alvarez-Tunon and Balaguer [37], using the UUV simulator (Gazebo Sim) to simulate underwater structures. The second one presents the results from Koreitem et al. [43], based in the environment from Aqua simulator [42]. The third sample shows the results from Yao et al. [46], used for the obstacle avoidance task. The fourth line brings the results from OpenWater [45], simulating realistic data for structures as well. The last one, HoloOcean [16], simulates remote sensing and navigation for underwater missions.

After developing a coherent imagery model [14], the researchers pointed their attention to other issues related to the subject. By leveraging parallel programming optimization and rendering frameworks, they released fully functional simulation environments [16,33,42,45]. These software allow other research teams around the world to produce relevant results without access to underwater real data or expensive sensors [16] to capture the information. Using these simulators, researchers can focus their efforts on generating particular datasets (high turbidity, structure captures, etc.) or task-related works (object detection and avoidance [46], vision-based navigation [16], etc.). Examples and results from these efforts can be seen in Figure 16.

However, even using these ready-to-use simulators, generating data to cover all the possible water types and environment coefficients is very expensive, in effort (programming or manual scene configuration) and time. Configuring the simulator, executing the program, navigating the scene, and capturing data must be repeated for other scenarios as many times as needed. A more efficient solution to augment datasets need to be implemented.

Artificial intelligence can be used to address this issue. In the timeline presented in Figure 7, the majority of advancements in recent years have been produced using some AI strategy. A simple machine learning method can learn a math model from the data. If a more complex model is required, a refined method is needed. An efficient solution to increase the model’s complexity is to add new layers, producing composed math functions to map the data. Because of that, we call them deep learning and deep models [77]. The CNNs use this strategy: a single convolution produces a math model, and multiple increase the complexity. Convolutions are originally from signal processing and are useful to interpret value change over time (2-dimensional processing). The behavior can be extended to retrieve information from images [78].

Thus, the feature extraction from convolutions was used by many researchers to classify data. The numeric representation of features produced can be useful for splitting data in N-dimensional space. In the selected papers, using Table 2 as reference, two publications [52,73] use this type of AI-model.

A useful strategy to address the augmentation task is style transfer [57]. Understanding the latent space (or learning the coefficients from this particular domain), the network can take an input and apply the learned space to produce a new output. For example, receiving a clean underwater capture as input, apply color shift distortion learned from real-world captures and produce a new sample degraded by color shift [17,32,34,52]. It is also possible to completely change the input image. To exemplify, the model can transform a common image, taken in the air, in an underwater sample [33,54,70]. At this point, the trained network requires more information to reach good results: the depth map from the input image, for example. The reason is the high dependency of capture distance and water depth in the underwater image formation model.

For now, it is possible to create synthetic data with high variability that represents different water types. However, many selected works in this review pointed to incrementing the realism level as a future improvement. If the training synthetic data do not represent real-world behavior, the laboratory-tested model would not be useful for in-the-wild captures. To increase the realism of produced images, adversarial training was presented: Given two models, one generator and one discriminator, they will compete trying to outsmart the other. The training will be executed until the generator produces images as realistic as the real-world dataset that the discriminator is unable to distinguish [19].

It is complex to develop a single generative model capable of addressing all relevant issues in underwater scenes. Consequently, some authors have created specialized models for each specific issue [59,66]. A valid method is to stack multiple generators to produce more degrading situations in a single pipeline [61,70].

However, adversarial networks are not limited to data generation. Synthetic images can be used to train generators to enhance underwater images [66,79,80]. The simulated data can validate the strategy, which will be refined later using real-world captures but without needing a huge amount of captures.

Nowadays, researchers understand the potential of improving the synthetic data using more than one rendering strategy. Each strategy has its pros and cons, but by combining them, we can minimize the impact of the disadvantages. Using the fast scenario assembly from the rendering framework to automate the image capture combined with the realism improvement from a generative model can produce a huge amount of data useful for various tasks [39,40]. Additionally, incorporating the light behavior fidelity of physics models before the AI improvement is also beneficial [73].

## 7. Further Directions

As mentioned before, a common future improvement presented is related to visual realism. Many authors identified generative models as a potential solution to enhance realistic perception. This concern can be related to other future research: high variability of water types in real-world data.

For any rendering strategy, mapping all the possible coefficients of water worldwide is complex. Suspended particles can change depending on the location, as can mineral presence, fauna, salt concentration, natural light incidence, and more. Many variables can influence underwater capture. This variation is important and can directly impact future works: real-world validation.

Most of the selected works (≈87%) tested their proposed process in controlled environments, using laboratory data or real-world data with low variability. These scenarios cannot represent the in-the-wild environment, tested only by ≈27%. The selected papers point to possible solutions, reproducing the real-world environment, but the weak validation data raise doubts about pipeline’s use for more complex configurations. However, recent research has yielded promising results using synthetic data to train models in other fields such as medicine [81] and autonomous vehicles [82].

Thus, we summarize the most common further improvements mentioned by authors in the selected papers:

- Visual Realism: increase the realism of the produced data and allow the synthetic images to be used as real-world data sources;
- High Variability: Pipelines with scene configuration enable researchers to generate large datasets efficiently;
- Real-world validation: produced models are not validated in real-world captured data.

From the future improvements, we can deduce four relevant characteristics that impact the result for each rendering strategy. Traditional rendering is highly dependent on water-type coefficients because these values are used in the computation, leading to high computational costs. Using the game engine rendering, the programming effort can be minimized, but the coefficients are still necessary. Finally, the AI models reduce dependency on coefficients, as they can be inferred from data. However, depth and distance information are required for most of the strategies to reach a realistic result. The realism achieved by each strategy varies depending on the requirements and available resources. Physics-based codes, such as physically based rendering [83], can produce realistic images but at a high computational cost. AI-based codes can also achieve high-fidelity images, as demonstrated by [20], but are limited to the trained environment. A comparison is presented in Table 3.

## 8. Conclusions

Recent advances in underwater research and the use of remotely operated vehicles in the ocean reveal the need for image processing tasks for underwater missions. For any vision-based tasks, such as object detection, navigation, or obstacle avoidance, quality images are required. Thus, the issues caused by water light behavior need to be considered to provide an enhanced capture.

However, validating any image enhancement algorithm requires access to relevant data, whether real-world or closely resembling it. Capturing such data is challenging due to the complexities of the environment. A viable approach to overcoming this limitation is the use of synthetic data, which can be generated through two primary methods: physics-based models and AI models.

The first models, based on physics, are used to estimate the light behavior in the underwater environment, increasing the realism in the generated images. The second one uses AI to understand the light behavior from examples and reproduce, creating new samples. Since the evolution of the AI techniques and computational power, the research teams migrate to this methodology. However, authors presented solutions using a combination of both methods: using the physics models to train the AI models, resulting in more accurate generated images.

Future improvements should focus on increasing the realism of the produced synthetic data and, most importantly, validating the models using real-world and complex data. Other areas of interest include controlled degradation features, using known measurement systems: Jerlov water type variations for the same scenario, turbidity levels using the ISO 7027-1:2016 scale, color shift variation for the same scenario, and the inclusion of artificial lights in the scenario.

## Figures and Tables

**Table 1:** Computer graphics paper comparison based on the features presented. The sign √ means the presence of this feature in the publication. ⌀ means not. If no information was found, we used -. “Def. Render.” and “Param. Render.” mean default rendering and parametrized rendering from the used platform.

#	Render	Technique	Real Time	Realism	Participant Media	Deep Water	Godrays	Haze	Color Shift
[23]	OpenGL	FFT, Navier– Stokes, Cube Map	√	⌀	⌀	⌀	√	√	⌀
[24]	OpenGL	Color blending, shadow mapping	√	-	⌀	⌀	√	√	⌀
[26]	OpenGL	Discrete Ordinates	√	⌀	√	⌀	⌀	⌀	⌀
[25]	-	Color blending, shadow mapping	√	⌀	⌀	⌀	√	√	⌀
[28]	-	FFT, Beer– Lambert, OSOA	√	⌀	√	√	⌀	⌀	√
[15]	OSG	-	-	⌀	⌀	⌀	⌀	⌀	⌀
[21]	Gazebo, osgOcean	-	√	⌀	-	⌀	√	√	√
[29]	-	-	√	√	√	⌀	⌀	⌀	⌀
[30]	Matlab	FFT, Beer–Lambert, FlourIS	√	-	⌀	⌀	⌀	⌀	⌀
[31]	Matlab	Jaffe–McGlamery, Ray Tracing	√	√	⌀	⌀	⌀	⌀	√
[35]	Gazebo, Unity	Def. Render.	√	⌀	-	√	⌀	⌀	⌀
[36]	Gazebo	Def. Render.	√	√	⌀	⌀	⌀	⌀	√
[42]	Unreal	Def. Render.	√	√	√	√	⌀	√	√
[43]	Unreal	Def. Render.	√	√	√	√	⌀	√	√
[44]	-	Ray Marching	√	⌀	⌀	√	√	√	-
[37]	Gazebo	Jaffe–McGlamery	√	⌀	⌀	√	⌀	⌀	√
[32]	-	-	√	⌀	⌀	⌀	⌀	⌀	√
[45]	Unreal	Ray Tracing, Def. Render.	√	√	⌀	⌀	⌀	√	⌀
[20]	Blender	Ray Tracing	-	√	⌀	√	⌀	⌀	√
[33]	-	-	√	⌀	⌀	√	⌀	⌀	√
[16]	Unreal	Param. Render.	√	√	-	√	⌀	√	√
[49]	Unity	Jaffe–McGlamery Foggy	√	√	⌀	√	⌀	√	√
[38]	Gazebo	Def. Render.	√	⌀	⌀	⌀	⌀	⌀	⌀
[50]	Unity	Def. Render.	√	-	⌀	⌀	⌀	√	⌀
[34]	-	-	√	⌀	⌀	⌀	⌀	⌀	√
[39,40]	Blender	Path-tracing	√	√	⌀	⌀	⌀	⌀	⌀
[46]	Unreal	Param. Render.	√	√	-	√	⌀	√	√
[47]	Unreal	Def. Render.	√	√	-	√	√	√	√

**Table 2:** AI Paper comparison based on the features presented. The sign √ means the presence of this feature in publication. ⌀ means not. If no information was found, we used -.

#	Model	GenAI	Style Transfer	Part. Media	Validation		Color Shift	Require Depth	Phys. Loss	Jerlov Types
					Real Data	In the Wild				
[61]	WaterGAN	√	⌀	-	√	√	√	√	⌀	-
[62]	Residual, VGG16	√	⌀	⌀	√	⌀	⌀	⌀	⌀	⌀
[70]	CycleGAN, conditional GAN	√	√	√	⌀	⌀	√	⌀	⌀	⌀
[63]	WassersteinGAN, patchGAN	√	⌀	⌀	√	⌀	√	⌀	⌀	⌀
[51]	Residual, DNN	⌀	√	⌀	√	⌀	√	√	⌀	√
[56]	ResNet, VAE	⌀	⌀	⌀	√	⌀	⌀	⌀	⌀	⌀
[57]	UWCNN	⌀	⌀	⌀	√	√	√	√	⌀	√
[32]	-	⌀	⌀	⌀	√	√	√	√	⌀	√
[66]	IPMGAN	√	⌀	⌀	√	√	⌀	⌀	√	⌀
[58,59]	Conditional GAN	⌀	√	⌀	√	⌀	√	√	√	√
[64]	Multiple GANs	√	√	⌀	√	⌀	√	√	-	⌀
[9]	GAN	√	⌀	⌀	√	⌀	⌀	⌀	⌀	⌀
[55]	-	⌀	⌀	⌀	√	⌀	√	-	-	√
[72]	Statistical Model	⌀	⌀	⌀	⌀	⌀	√	⌀	⌀	√
[71]	VGG	⌀	√	⌀	√	⌀	⌀	⌀	⌀	⌀
[52]	CNN	⌀	√	⌀	√	√	-	⌀	⌀	⌀
[73]	CNN	⌀	√	⌀	⌀	⌀	-	⌀	⌀	⌀
[53]	CycleGAN	√	√	⌀	√	√	-	⌀	⌀	⌀
[54]	Multiple GANs	√	√	√	√	⌀	-	-	√	⌀
[60]	MLP	⌀	√	√	√	⌀	√	⌀	√	⌀
[68]	GAN	√	⌀	√	√	⌀	⌀	⌀	⌀	⌀
[67]	Submerge StyleGAN, VGG19	√	√	⌀	√	⌀	⌀	√	⌀	⌀

**Table 3:** Relevant Features and their impact on output data. Green cells represent an advantage. Otherwise, red and orange represent a disadvantage.

Rendering Strategy	Water-Type Coeff. Dependency	Depth and Distance	Computational Cost	Programming Effort	Realism
Physics	High	High	High	High	Low
Rendering Frameworks	High	High	Optimized	Low	Medium
Artificial Intelligence	Low	High	Low	Optimized	High
